# Combined Return Synergizes Aboveground Photosynthesis and Subsurface Soil Organic Carbon Stock to Boost Spring Wheat Yield in the Qinghai–Tibet Plateau

**DOI:** 10.3390/plants15192909

**Published:** 2026-09-23

**Authors:** Xiaoyan Wang, Nannan Hu, Mingdan Song, Mei Han, Zhengpeng Li

**Affiliations:** 1 Academy of Animal Science and Veterinary, Qinghai University, Xining 810016, China; 18409481505@163.com (X.W.); hu733n@163.com (N.H.); 2 Academy of Agriculture and Forestry Sciences, Qinghai University, Xining 810016, China; hi0045@163.com (M.S.); hanmei20061234@sina.com (M.H.)

**Keywords:** material return, carbon fixation and carbon metabolism functional genes, carbon storage, photosynthesis, yield

## Abstract

The Qinghai–Tibet Plateau is a key component of global plateau ecosystems, but the synergistic yield-enhancing mechanism integrating aboveground photosynthetic productivity and belowground increase in soil organic carbon stock under combined green manure and wheat straw incorporation remains unclear. Based on a field experiment established in 2021, four treatments were designed (green manure return (G), straw return (W), combined return (GW), and no-return control (N)) to systematically investigate the effects of return methods on the photosynthetic characteristics, carbon-cycling functional genes, and grain yield of spring wheat. All return treatments significantly increased spring wheat yield, dry matter accumulation, and yield components, with GW showing the greatest effect. The return treatments also increased the net photosynthetic rate, stomatal conductance, transpiration rate, and chlorophyll content, while decreasing intercellular CO_2_ concentration. Straw return improved bacterial community evenness; the GW treatment increased microbial β-diversity, enriched Chloroflexi and *Fusarium*, and constructed a functionally complementary microbial community. Green manure return increased the community richness of the cbbS gene, while straw return reduced the community evenness of the *ppdK* gene; different return materials directionally reshaped carbon-cycling microbial communities. rbcL was predominantly affiliated with *Bradyrhizobium*; *korA, ppdK, and sdhA* were mainly hosted by *Nocardioides*; *Amaricoccus* was the dominant genus carrying *cbbS*, while *Flavobacterium* prevailed in communities harboring *accA* and *gapA*. Co-occurrence network analysis revealed that green manure or straw return alone formed compact networks with high clustering coefficients and short path lengths, dominated by Rokubacteriota and Dormibacterota; the GW network exhibited lower average degree and shorter path length, with Saccharimonadales replacing the original dominant taxa to form a modular structure characterized by low redundancy and high efficiency. Soil organic carbon stock increased under all treatments, with the greatest increment observed under GW. In summary, combined green manure and wheat straw return enhances spring wheat yield by strengthening aboveground photosynthetic performance and optimizing yield components, while simultaneously activating belowground carbon-cycling functional genes, restructuring microbial co-occurrence networks, and promoting soil organic carbon accumulation.

## 1. Introduction

As the second major grain crop following highland barley (*Hordeum vulgare* L.) on the Qinghai–Tibet Plateau, wheat (*Triticum aestivum* L.) occupies one-third of the total grain sown area [1]. It is also widely cultivated with high yield potential worldwide, playing a vital role in food security guarantees [2]. Crop straw as main agricultural by-products and traditional treatment methods lead to resource waste and environmental pollution [3]. Farmers in this region typically use part of the wheat straw as feed or fuel, and part of the straw is removed from the field. Previous studies have demonstrated that the introduction of green manure via multiple cropping or rotation practices can boost the growth of cereal crops, enhance the accumulation of photosynthetic assimilates, and facilitate the efficient translocation of assimilates to grains during the late growth stage, thereby improving crop yield [4,5,6]. Leguminous green manure possesses prominent nitrogen conservation capacity owing to symbiotic nitrogen fixation with rhizobia [7]. Nevertheless, leguminous green manure features a relatively low C/N ratio [8]. Its rapid decomposition and mineralization after soil incorporation tend to trigger nutrient loss and stimulate soil microorganisms to deplete native soil organic carbon [9]. By contrast, gramineous straw has a high C/N ratio [10]. Straw incorporation elevates soil organic carbon content and ameliorates soil physicochemical properties, which further facilitates crop development [11]. Nonetheless, substantial nitrogen is consumed by soil microorganisms during straw decomposition, giving rise to nitrogen competition between microbes and crops [12]. Accordingly, the combined incorporation of leguminous green manure and gramineous crop straw can better regulate the C/N ratio of returned organic materials and achieve superior soil fertility improvement relative to separate single-material returning, thereby effectively promoting crop yield enhancement [8]. The combined application of straw and green manure incorporation coupled with chemical fertilizer reduction can effectively increase spike number and grains per spike, thereby improving wheat yield [13]. Additionally, green manure multiple cropping with appropriate nitrogen reduction after wheat harvest enhances the spike density, grains per spike and 1000-grain weight of wheat, contributing to a 12.4% increase in grain yield [14]. Consequently, the integration of multifunctional field practices, including straw–green manure combined returning and nitrogen fertilizer reduction, serves as a critical strategy to ameliorate soil quality and achieve sustainable, stable and high crop yields.

Photosynthesis serves as a fundamental physiological process whereby crops assimilate inorganic substances into organic matter and store energy. The magnitude of photosynthetic capacity is critical for sustaining stable and high crop yields. In addition to climatic factors, soil water and nutrient availability substantially regulate crop photosynthetic performance [15]. Organic residue incorporation can loosen soil structure, improve soil permeability, expand crop photosynthetic area, and facilitate crop dry matter accumulation [16]. A higher photosynthetic rate enables greater organic matter assimilation in plants, which substantially benefits crop yield formation [17]. Ran et al. [18] reported that straw incorporation could elevate stomatal conductance, which further enhanced photosynthetic rate. Straw return firstly improves soil hydrothermal conditions, nutrient availability and microbial activity, thereby promoting root nutrient uptake, optimizing leaf nitrogen status and chlorophyll synthesis, and ultimately affecting net photosynthetic rate, transpiration rate, stomatal conductance and other photosynthetic parameters [19]. Chlorophyll acts as an indispensable substance for photosynthesis in green plants, and its content directly governs plant photosynthetic capacity [20]. Straw return mainly affects crop root nutrient uptake, leaf nitrogen status, chlorophyll synthesis, and photosynthetic performance indirectly by improving soil hydrothermal conditions, enhancing nutrient availability, and regulating microbial activity. In addition, straw mulch on the soil surface can further affect photosynthesis via pathways such as light reflection and the regulation of soil temperature and moisture conditions. Previous studies have demonstrated that straw incorporation delays chlorophyll degradation in wheat leaves and elevates chlorophyll content. These improvements further boost net photosynthetic rate, transpiration rate and stomatal conductance, and ultimately increase spike number, grains per spike, 1000-grain weight, grain yield and biomass yield [21,22].

Farmland soil carbon stock serves as a core indicator of soil quality and fertility, and profoundly regulates agricultural sustainable development and global food security. Soil microorganisms act as the pivotal drivers governing the transformation and accumulation of soil organic carbon [23]. Straw incorporation elevates the abundance and diversity of soil bacteria and fungi. Copiotrophic taxa including Proteobacteria and Bacteroidetes predominate in bacterial communities, which possess strong capacity to utilize labile organic carbon. The rising abundance of Basidiomycota and partial Ascomycota in fungal communities indicates enhanced decomposition potential of cellulose and lignin within incorporated straw residues [24,25]. When double-cropping rice straw return, the relative abundance of bacteria and fungi decreases, but when single-cropping rice straw return it increases [26]. In different regions, the effects of straw return on fungal diversity vary [27].

Functional genes related to carbon sequestration play a critical role in sustaining soil organic carbon stability [28]. The Calvin–Benson–Bassham (CBB) cycle constitutes the most important carbon fixation pathway [29]. RubisCO is the rate-limiting enzyme of this pathway, encoded by *cbbL* and *cbbS*, which correspond to the large and small subunits of Type I RubisCO, respectively [30], whereas *rbcL* is widely distributed in autotrophic microorganisms and rhizospheric carbon-fixing bacteria [31]. *gapA* encodes another core CBB enzyme that catalyzes the reduction of 3-phosphoglycerate. In addition to the CBB cycle, farmland microorganisms utilize alternative pathways [32], including *accA*, *sdhA* and *korA*, *por*, and *ppdK* [33,34,35,36].

The soil microbial community serves as a key biological link connecting soil carbon and nitrogen transformations and crop productivity. Combined return of green manure and straw inputs readily decomposable carbon and nitrogen substrates, and alters the composition and function of soil microbial communities, thereby promoting nutrient mineralization and available nitrogen supply, enhancing crop root uptake and leaf photosynthetic capacity, and ultimately increasing dry matter accumulation and grain yield. However, in the alpine croplands of the Qinghai–Tibet Plateau, the cascade relationship among soil, microbiome, plant and yield under combined return of green manure and wheat straw remains to be further elucidated. Relevant studies on the Qinghai–Tibet Plateau mostly focus on the yield-increasing effects of single material return or its impacts on individual aboveground or belowground systems; the aboveground–belowground synergistic mechanism driving wheat yield gain under combined residue return remains unclear.

Against this background, this study was conducted based on a multi-year field experiment on the Qinghai–Tibet Plateau. Three field management practices, including straw incorporation, green manure incorporation, and their combined incorporation, were selected as the research treatments. Key indicators, encompassing photosynthetic parameters, dry matter accumulation, yield components, carbon sequestration functional genes, and soil organic carbon stock, were systematically determined. This study aims to clarify the mechanistic pathway by which combined organic incorporation promotes spring wheat yield enhancement through bidirectional effects between aboveground photosynthetic productivity and belowground increase in soil organic carbon stock capacity. These findings provide a theoretical basis for improving spring wheat yield on the Qinghai–Tibet Plateau through the coordination of aboveground photosynthetic productivity and the increase in belowground soil organic carbon stock in regional plateau farmlands, and offer scientific support for the sustainable management of farmlands on the Qinghai–Tibet Plateau.

## 2. Materials and Methods

### 2.1. Study Site Description

This study was based on a field plot experiment initiated in 2021 at the experimental field of the Academy of Agriculture and Forestry Sciences, Qinghai University, Chengbei District, Xining City, Qinghai Province (36°43′22″ N, 101°44′57″ E). The site is at an elevation of 2314 m, with a plateau continental semi-arid climate; the mean annual temperature is 6.37 °C and the mean annual precipitation is 475.55 mm (Figure 1). The soil type is chestnut soil. The basic soil physicochemical properties before the experiment are shown in Table 1.

### 2.2. Experimental Design

The field experiment was established in 2021. Soil samples for organic carbon determination were collected at the wheat harvesting stage annually from 2022 to 2025, grain yield was sampled from 2023 to 2025, and other indicators (net photosynthetic rate, stomatal conductance, transpiration rate, intercellular CO_2_ concentration, chlorophyll content, and soil metagenome) were sampled in 2025. The four treatments were established as follows: green manure incorporation (G), in which only green manure and its root stubble were incorporated without wheat straw addition; wheat straw incorporation (W), in which aboveground straw together with root stubble were fully incorporated; combined incorporation (GW), in which wheat aboveground straw, wheat root stubble, green manure, and its root stubble were all incorporated together; and no incorporation (N), in which wheat straw was removed, serving as the control. All residue treatments were applied as full incorporation. Each treatment was performed in triplicate, resulting in 12 plots in total, with each plot covering an area of 16 m^2^ (4 m × 4 m). In this experiment, nitrogen fertilizer was applied as urea (containing 46% N) and diammonium phosphate (containing 18% N), with a nitrogen application rate of 135 kg N·ha^−1^. Nitrogen fertilizer was split into two applications: basal application before sowing (80%) and topdressing at the four-leaf stage (20%), at a basal-to-topdressing ratio of 4:1. After wheat harvest, in the green manure (hairy vetch, *Vicia villosa* Roth) return treatments, hairy vetch was sown in mid-to-late August at a seeding rate of 150 kg·ha^−1^. At the full-bloom stage (early-to-mid October), the green manure was crushed together with wheat straw (to ≤20 cm) and incorporated into the soil. During the crop growing season, irrigation was applied three times in mid-May, mid-June and early November, with 80 mm applied each time. Prior to the establishment of the experiment, the preceding crop was spring wheat. Other field management practices followed local conventional standards.

### 2.3. Sample Collection and Processing

#### 2.3.1. Soil Sampling

Soil organic carbon: Soil samples were collected from the 0–20 cm soil layer using a soil auger via the S-shaped five-point sampling method at the wheat harvesting stage in 2022–2025. Each treatment had three biological replicates, all samples were collected separately from each replicate plot, and the samples were used for the determination of soil organic carbon and soil metagenome. Soil bulk density was measured using the core method. At the wheat harvest stage each year from 2022 to 2025, undisturbed soil cores (100 cm^3^ stainless steel rings) were collected from the 0–20 cm soil layer using an S-shaped five-point sampling strategy in each plot. Three independent replicates were sampled per plot. All soil samples were oven-dried at 105 °C to constant mass before weighing.

#### 2.3.2. Yield and Yield Components

Dry matter accumulation: At wheat maturity, ten representative plants were sampled from each plot. Plants were blanched in an oven at 105 °C for 30 min and then oven-dried at 80 °C to constant weight before measuring plant dry weight. The average dry weight of the ten plants per plot was regarded as the crop dry matter accumulation for that plot. Each treatment included three replicate plots (i.e., three independent replicates). The mean value of the three plots was used as the crop dry matter accumulation of the corresponding treatment for statistical analysis.

Yield components: At the spring wheat maturity stage in 2025, wheat plants within a one-meter row length were randomly sampled from each plot for grain yield, spike number, grains per spike, and 1000-grain weight.

Grain Yield: At the maturity stage of spring wheat during 2023–2025, the border rows of each plot were discarded to minimize marginal effects. Plants within each plot were harvested and threshed separately to determine the actual grain yield, which was subsequently converted to yield per hectare. Grain yield was measured annually in 2023, 2024, and 2025. Data were statistically analyzed on an annual basis, and results are presented as separate yearly analyses instead of a three-year mean.

### 2.4. Measurement Items and Calculation Methods

#### 2.4.1. Measurement Items

1. Determination of photosynthetic characteristics of wheat flag leaves. During the grain-filling stage of wheat, the net photosynthetic rate (Pn), stomatal conductance (Gs), transpiration rate (Tr), and intercellular CO_2_ concentration (Ci) of flag leaves were measured using a portable photosynthetic system (LI-6400XT, LI-COR Biosciences, Lincoln, NE, USA). Measurements were performed on sunny days from 09:00 to 11:00. For each plot, ten leaves were sampled and measured. The measurements from the ten leaves were first averaged to obtain a single plot-level mean, which was then used as the experimental observation in all statistical analyses. Thus, the plot, rather than individual leaves or subsamples, was treated as the experimental unit, with *n* = 3 independent replicates per treatment.
2. Determination of relative chlorophyll content in wheat flag leaves. During the wheat grain-filling stage, the relative chlorophyll content of flag leaves was measured on sunny days from 09:00 to 11:00 using a portable chlorophyll meter (SPAD-502, Konica Minolta, Osaka, Japan). Ten uniform and healthy flag leaves were randomly selected per plot, and the average SPAD readings were recorded to represent leaf chlorophyll status.
3. Soil organic carbon (SOC) was determined via the potassium dichromate external heating method [37].
4. Soil pH was measured via the potentiometric method at a water-to-soil ratio of 2.5:1, with deionized water as the extraction medium, using an Orion 868 pH meter.
5. Soil microbial DNA extraction and sequencing.

Total soil DNA was extracted using the Fast DNA^®^ Spin Kit (MP Biomedicals, Solon, OH, USA) for soil. The qualified genomic DNA was detected via 1% agarose gel electrophoresis and subsequently sheared into fragments of approximately 350 bp using a Covaris M220 ultrasonicator (Covaris, Woburn, MA, USA). After ligation with Y-shaped adapters, magnetic bead screening was performed to eliminate self-ligated adapter fragments. The library templates were enriched by PCR amplification to construct paired-end (PE) libraries. Finally, bridge PCR amplification was conducted prior to high-throughput sequencing. All metagenomic sequencing procedures were performed by Shanghai Majorbio Bio-Pharm Technology Co., Ltd., Shanghai, China. Raw sequencing reads were filtered by trimming adapters, low-quality sequences and host contaminants to acquire high-quality clean data. The non-redundant gene set was aligned against the NR database using DIAMOND software (version 2.1.11). BLASTP algorithm (Version 2.2.28+) was adopted with the e-value cutoff set at 1 × 10^−5^. Species annotation was performed according to the NCBI Taxonomy database matched with the NR database. Sequences affiliated with Bacteria and Fungi were separately extracted for subsequent microbial community analysis. Predicted gene sequences were further annotated against the KEGG database to obtain KEGG Orthology profiles. Genes associated with soil carbon sequestration were screened to analyze carbon-fixation-related functional genes and their corresponding microbial communities.

#### 2.4.2. Calculation Methods

Soil organic carbon stock [38]

SOCs = SOC × BD × h × 10

SOCs is the soil organic carbon stock (t·ha^−1^); SOC is the organic carbon concentration of each soil layer (g·kg^−1^); BD is soil bulk density (g·cm^−3^); and h is soil layer thickness (m).

### 2.5. Data Processing

Data sorting was conducted using Microsoft Excel 2016. Statistical analyses were performed using SPSS 22.0 and R 4.0.2. Prior to parametric tests, all data were examined for normality using the Shapiro–Wilk test and for homogeneity of variances using Levene’s test. The plot served as the experimental unit (*n* = 3), with within-plot subsamples averaged prior to statistical analysis. For data that met both assumptions of normality and homogeneity of variance, one-way analysis of variance (ANOVA) was performed to evaluate overall treatment effects. When a significant F-test was detected (*p* < 0.05), Duncan’s multiple-range test was subsequently applied for pairwise comparisons. For data that did not meet the assumptions of normality or homogeneity of variance, the Kruskal–Wallis rank-sum test was employed to assess overall differences among treatments. When the Kruskal–Wallis test was significant (*p* < 0.05), Dunn’s test was used for post-hoc pairwise comparisons, with *p*-values adjusted using the Bonferroni correction to control the family-wise error rate. A significance level of *p* < 0.05 was considered statistically significant in all analyses. Different letters in figures indicate significant differences among treatments (*p* < 0.05). All figures were plotted and visualized using Origin 2024. Based on Bray–Curtis distances, non-metric multidimensional scaling (NMDS) analysis was performed using R 4.0.2 to analyze the β-diversity of bacterial, fungal, and carbon-sequestration-gene-carrying microbial communities, aiming to elucidate the influence of residue incorporation on the community structure of carbon-sequestration genes. To explore the interactions among microbial taxa, co-occurrence networks were constructed. Pairwise correlations were calculated using Spearman’s rank correlation coefficient. Significant correlation pairs satisfying ∣r∣ > 0.6 and *p* < 0.05 were retained for network construction. Each network was constructed based on 3 samples (three biological replicates per treatment, with a total of 12 samples). Network topological parameters were calculated and visualized using Gephi 0.10.1. The Mantel test was performed using the linkET package in R (v. 4.0.2). A random forest model was applied to identify the key factors driving crop yield variation under straw residue return practices. The model was established based on 12 independent plot-level samples, with predictive variables encompassing photosynthetic parameters, carbon fixation and carbon metabolism functional genes, carbon stock, dry matter content, soil organic carbon, as well as fungal and bacterial communities. Model construction was performed using the randomForest package in R 4.0.2, with the number of trees set to 500. Variable importance was quantified based on the percentage increase in mean squared error, and model performance was validated using the out-of-bag error.

## 3. Results

### 3.1. Crop Yield, Yield Components and Dry Matter Accumulation Under Straw Returning

Dry matter accumulation, grain yield and yield components were all enhanced by straw returning, and combined incorporation yielded the optimal effect (Figure 1). The average wheat yield was 6038.62 kg·ha^−1^ (Figure 1c). Under different material returnings, compared with N, the grain yields of GW, G, and W increased by 30.44%, 23.06%, and 10.59% (*p* < 0.05); the dry matter accumulation increased by 34.19%, 15.81%, and 18.22% (*p* < 0.05) (Figure 1a). Under different straw returning treatments, the treatments N, GW, G and W all improved yield components (Figure 1b). Specifically, grain number per spike increased by 24.86%, 6.99% and 14.74% (*p* < 0.05); spike number increased by 21.30%, 19.38% and 13.60% (*p* < 0.05); and 1000-grain weight increased by 34.98%, 29.03% and 20.75% (*p* < 0.05).

### 3.2. Leaf Photosynthetic Physiological Indicators Under Material Returning

Straw returning increased leaf net photosynthetic rate, stomatal conductance, transpiration rate and relative chlorophyll content of wheat. Compared with treatment N, the net photosynthetic rate of leaves in treatments GW, G and W increased by 10.83%, 6.35% and 4.11% (*p* < 0.05) (Figure 2a). Leaf stomatal conductance rose by 7.53%, 4.62% and 2.62% (*p* < 0.05) (Figure 2b), leaf transpiration rate by 4.20%, 3.00% and 1.52% (*p* < 0.05) (Figure 2c), and SPAD values by 66.67%, 41.34% and 42.54% (*p* < 0.05) (Figure 2e). Straw returning reduced leaf intercellular CO_2_ concentration. Compared with the control, treatments GW, G and W caused respective decreases of 3.04%, 2.02% and 1.12% (*p* < 0.05) (Figure 2d).

### 3.3. Soil Organic Carbon and Its Storage Under Straw Returning

Over the period from 2022 to 2025, the mean soil organic carbon content and stock exhibited a progressive increase across the treatments of hairy vetch incorporation, straw incorporation, and their combined application (Figure 3). Residue return had no significant effect on soil bulk density (Figure S2). Therefore, differences in organic carbon among treatments were mainly driven by changes in SOC concentration, rather than by changes in bulk density. Relative to the N treatment, the mean organic carbon stocks under GW, G, and W were elevated by 37.65%, 19.46%, and 17.96%, respectively (*p* < 0.05) (Figure 3b), with the GW treatment yielding the highest values for both organic carbon content and stock.

### 3.4. Soil Bacteria and Fungi Under Straw Returning

#### 3.4.1. Variations in Bacterial and Fungal Diversity

The bacterial Simpson index was markedly reduced under treatment W (Figure S3), reflecting lower bacterial community evenness. Several microbial taxa that efficiently degrade cellulose and hemicellulose were selectively enriched and turned into dominant species, whereas the relative abundance of most other bacteria decreased due to resource competition and environmental shifts. Ecologically, this simplified community structure is conducive to accelerated straw decomposition and nutrient turnover.

#### 3.4.2. NMDS Analysis of Bacterial and Fungal Communities

Non-metric Multidimensional Scaling (NMDS) analysis revealed that straw returning altered the structure of soil bacterial and fungal communities and induced distinct community differentiation (Figure S4). The NMDS results of fungal and bacterial communities were as follows: fungi (stress = 0.099; PERMANOVA: F = 1.53, R^2^ = 0.364, *p* = 0.025); bacteria (stress = 0.015; PERMANOVA: F = 3.67, R^2^ = 0.579, *p* = 0.58). The PERMDISP test revealed no significant differences in the within-group dispersion of fungal and bacterial communities across treatments. All permutation analyses were performed with 999 permutations. For the bacterial community, the greatest dissimilarity was observed between treatment GW and the non-returning control N, whereas W shared a relatively high similarity with N. For the fungal community, a gradual gradient differentiation was detected along the ordination axis from N to GW, and fungal community structures of G and W were highly similar. Overall, combined straw returning exerted the strongest effect on reshaping microbial community structure, and differed from single straw returning and the non-returning treatment. These findings demonstrate that organic material incorporation, particularly combined returning, can directionally regulate soil microbial communities in dryland farmland.

#### 3.4.3. Changes in the Abundances of Soil Bacteria and Fungi

Fungal community abundance showed marked differences among treatments (Figure S5). The GW treatment had the highest fungal relative abundance, which was greater than that of treatment N. In comparison, bacterial abundance varied slightly and remained stable across all treatments. Although GW and G had higher bacterial abundance than N, no significant differences were detected among groups.

#### 3.4.4. Changes in Soil Bacterial and Fungal Community Composition

The top 10 genera in terms of relative abundance were selected to visualize the compositions of bacterial and fungal communities (Figure S6). The predominant bacterial genera across all treatments included *Nocardioides*, *Luteolibacter*, *Gaiella*, unclassified_c_Candidate_Binatia and *Sphingobacterium*. The dominant fungal genera were *Aspergillus*, *Morchella*, *Fusarium*, *Colletotrichum* and *Trichoderma*.

#### 3.4.5. Differential Analysis of Soil Bacteria and Fungi

LEfSe analysis revealed that different straw returning methods significantly altered the composition of indicator species in soil bacterial and fungal communities (Figure 4). Treatment GW markedly enriched the bacterial phylum Chloroflexi and genus *Streptomyces*, as well as the fungal genera *Fusarium* and *Chaetomium*, thereby forming a microbial community with functional complementarity. Treatments G and W selectively enriched nitrogen-adapted microbiota and recalcitrant carbon-degrading bacteria, respectively, leading to obvious functional divergence of communities. In contrast, treatment N was predominated by oligotrophic microbes and exhibited a remarkable decline in functional diversity. These findings further verify that combined straw returning optimizes the soil microecological structure of dryland farmland via directional regulation of key indicator microorganisms.

#### 3.4.6. Co-Occurrence Network Analysis of Soil Bacterial and Fungal Communities

Co-occurrence network analysis revealed that both bacterial and fungal communities formed complex interactive networks, with significant differences in topological structures (Figure S7, Table S2). The bacterial network was more compact and highly connected. It had a higher average degree (26.76) than the fungal network (15.375), along with a smaller network diameter and a shorter average path length. These results suggest that bacterial communities possess higher efficiency in material and information transfer. Additionally, the bacterial network had a slightly higher average clustering coefficient (0.777) than the fungal network (0.754), while its modularity index (0.1) was lower than that of fungi (0.153). These traits imply that the bacterial network features stronger global connectivity and weaker modularity. *Candidatus Defluviilinea* and *Nitrospira* served as the core hubs in the bacterial network. By contrast, the fungal network was dominated by key nodes of *Fusarium* and *Mortierella*, accompanied by higher modularity. This highly connected and structurally complementary co-occurrence network indicates that organic material returning enhances the stability and functional complementarity of soil microbial communities.

### 3.5. Soil Carbon Fixation and Carbon Metabolism Functional Genes

#### 3.5.1. Variations in the Diversity of Carbon Fixation and Carbon Metabolism Functional Genes

Green manure returning and combined straw returning markedly altered the diversity of carbon fixation and carbon metabolism functional genes (Figure S8). The G treatment significantly elevated the Chao1 index of the *cbbS* gene. This suggests that green manure input increases labile carbon and nitrogen contents and expands ecological niches, facilitating the survival and proliferation of rare autotrophic carbon-sequestering taxa. Consequently, the species richness of functional microorganisms for carbon sequestration was greatly improved. Treatment W significantly decreased the Simpson index of the *ppdK* gene. This indicates that straw returning enabled a small number of taxa adapted to straw-derived carbon sources to become dominant, which in turn reduced community evenness and the Simpson index substantially.

#### 3.5.2. NMDS Analysis of Carbon Sequestration Gene Communities

To explore the effects of field experiment nitrogen addition on the community structure of nitrification and denitrification functional genes, we performed NMDS based on Bray–Curtis distance to visualize beta diversity differences across *cbbS*, *gapA*, *ppdK*, *sdhA*, *rbcL*, *korA* and *accA* gene communities (Figure S9). The NMDS results for each functional gene community were as follows: *accA* (stress = 0.109; PERMANOVA: F = 1.37, R2 = 0.339, *p* = 0.068); *por* (stress = 0.067; PERMANOVA: F =1.37, R^2^ = 0.307, *p* = 0.068); *rbcL* (stress = 0.171; PERMANOVA: F = 1.54, R^2^ = 0.366, *p* = 0.023); *sdhA* (stress = 0.083; PERMANOVA: F = 1.84, R^2^ = 0.409, *p* = 0.004); *cbbS* (stress = 0.139; PERMANOVA: F = 1.89, R^2^ = 0.415, *p* = 0.031); *korA* (stress = 0.079; PERMANOVA: F = 1.62, R^2^ = 0.378, *p* = 0.034); *ppdK* (stress = 0.075; PERMANOVA: F = 2.06, R^2^ = 0.436, *p* = 0.002); and *gapA* (stress = 0.096; PERMANOVA: F = 1.57, R^2^ = 0.37, *p* = 0.008). PERMDISP analysis showed no significant differences in the within-group dispersion of each functional gene community across treatments, and all permutation analyses were performed using 999 permutations. All treatments exhibited distinct clustering separation, indicating that different returning methods directionally shape the community structure of carbon-cycling functional microorganisms. The non-returning treatment showed low dispersion in box-and-whisker plots, whereas returning treatments presented greater variability, suggesting higher within-treatment compositional heterogeneity. These findings demonstrate that organic material returning alters the spatial stability of carbon-cycling microbial communities.

#### 3.5.3. Changes in the Abundance of Carbon Fixation and Carbon Metabolism Functional Genes

Organic material returning elevated the abundance of carbon fixation and carbon metabolism functional genes (Figure S10). Straw returning significantly increased the abundance of the *por* gene by 33.80% (*p* < 0.05), while combined returning enhanced the *cbbS* gene abundance by 46.71% (*p* < 0.05).

#### 3.5.4. Changes in Community Composition of Carbon Fixation and Carbon Metabolism Functional Genes

We selected the top 10 genera in terms of relative abundance to profile the composition of carbon sequestration gene communities (Figure S11). Across all treatments, *Bradyrhizobium* dominated the *rbcL* community and *Nocardioides* was the predominant genus for *korA*, *ppdK* and *sdhA*. The dominant taxa were unclassified_c_Candidate_Binatia for *por, Amaricoccus* for *cbbS*, and *Flavobacterium* for *accA* and *gapA*.

#### 3.5.5. Differential Analysis of Carbon Fixation and Carbon Metabolism Functional Genes

Significant differences in the relative abundance of the *korA* gene were observed for phyla Chloroflexota, Candidatus_Deferrimicrobiota, Candidatus_Margulisbacteriota and Candidatus_ Lindowiibacteriota across all treatments (Figure 5). For the *ppdK* gene, significant differences were detected across all treatments in phyla Actinomycetota, Pseudomonadota, Candidatus_Binatota, Rhodothermota, candidate_division_WOR-3, Candidatus_Tectimicrobiota and Bdellovibrionota. For the *gapA* gene, significant differences were observed across all treatments in phyla Actinomycetota, Candidatus_Binatota, Candidatus_Methylomirabilota and Candidatus_ Deferrimicrobiota. Significant differences in the *sdhA* gene were detected across all treatments for the phylum Actinomycetota. For the *rbcL* gene, significant differences were found across all treatments in Picozoa and unclassified_d_Eukaryota. Significant differences in the *accA* gene were observed across all treatments for the phylum Verrucomicrobiota.

#### 3.5.6. Co-Occurrence Network Analysis of Carbon-Sequestration-Related Gene Communities

Network analysis was applied to explore shifts in key carbon-sequestering bacterial taxa under different organic returning practices (Figure S12, Table S2). The co-occurrence network revealed that treatments W and G had higher average clustering coefficients and shorter average path lengths. Their dominant bacteria belonged to uncultured taxa of Rokubacterota and Dormibacterota. These results suggest that the microbial communities under W and G formed highly modular and closely connected networks. By contrast, treatment N exhibited the largest network diameter and the longest average path length, indicating its network structure was the most extended and loosely connected. Compared with treatment N, GW presented a smaller network diameter, higher average clustering coefficient, shorter average path length and lower average degree. Its dominant bacterial group was *Saccharimonadales*. These findings suggest that the network of GW was more compact with stronger local clustering and higher efficiency of information transmission. Meanwhile, the average number of connections per node decreased, forming an efficient and highly modular network structure.

### 3.6. Correlations of Bacterial and Fungal Communities with Carbon Fixation and Carbon Metabolism Functional Genes and Crop Yield

Mantel test results (Figure 6) revealed that the *gapA* gene was significantly positively correlated with SOCs, Pn, Ci, Gs, Tr, SPAD and DM (*p* < 0.01). The *ppdK* gene showed positive correlations with Ci, Tr and SPAD (*p* < 0.05). Additionally, bacterial communities were positively associated with Tr (*p* < 0.05) (Figure 6a). Soil properties, photosynthetic indices and yield were all significantly positively correlated (*p* < 0.01). Random forest analysis indicated that Ci, *korA* and SOCs acted as the key drivers of yield (*p* < 0.01) (Figure 6b).

## 4. Discussion

### 4.1. Effects of Organic Material Returning on Crop Yield and Photosynthetic Characteristics

Previous studies have demonstrated that organic material return releases nitrogen, phosphorus, potassium and other nutrients during decomposition, which facilitates the growth of subsequent crops, and that sufficient soil nitrogen is a prerequisite for high crop yields [39]. However, since the present study did not directly measure soil nutrient contents and the experimental period was limited, this statement is provided only as background for discussion rather than as a main finding of this study. Partial substitution of chemical nitrogen fertilizer with green manure can improve soil nitrogen supply and enhance crop nutrient use efficiency, thereby increasing crop yield [40]. Studies by Zhang et al. [41] also demonstrated that organic material returning can partially substitute chemical fertilizers while maintaining stable crop yield. Compared with the separate return of green manure and crop straw, their combined incorporation improves soil aggregate stability and microbial activity [13,42]. The present study revealed that all material returning treatments increased wheat yield relative to the non-returning control, with combined returning showing the best performance (Figure 1c). The co-application of straw with a high C/N ratio and green manure with a low C/N ratio can regulate the C/N ratio of mixed residues to a range favorable for microbial decomposition. This practice alleviates microbial nitrogen competition induced by sole straw incorporation and compensates for insufficient nutrient supply at the late growth stage under single green manure application, ultimately improving crop yield. Zhao et al. [5] also reported that straw and green manure represent clean organic nutrient sources. The combined incorporation of straw, green manure, and chemical fertilizers increases the spike number and grains per spike, thereby promoting wheat yield formation. Growing green manure in the wheat rotation system combined with moderate nitrogen reduction increased wheat grain yield by 12.4%, mainly by improving spikes per unit area, grains per spike and 1000-grain weight [14]. Under the combined incorporation of Chinese milk vetch and rice straw, reducing nitrogen fertilizer input by 20–40% can maintain or even improve crop yield [43]. This finding is consistent with our observation that the GW treatment produced the greatest values for spikes per unit area, grains per spike, 1000-grain weight and grain yield in this study (Figure 1b). The magnitude of dry matter accumulation can comprehensively reflect the functional strength of the crop photosynthetic source and the conversion efficiency of photosynthetic products, plays a decisive role in the formation of wheat yield, and is a focus in the study of high-yield mechanisms [44]. The present study showed that all organic returning treatments increased crop dry matter accumulation, with combined returning exhibiting the best performance (Figure 1c). Exogenous organic material incorporation elevates soil organic carbon content (Figure 3a), facilitates the translocation and storage of photosynthetic products, and ultimately promotes plant dry matter accumulation. Furthermore, combined returning balances the dry matter accumulation rate and duration across the whole growth period, thereby achieving the maximum dry matter accumulation.

Wheat grain yield formation mainly depends on two carbon source pathways: one is the direct transport of photosynthetic products to the grains, and the second is the transfer of carbohydrates accumulated in vegetative organs such as stems and leaves to the grains. Therefore, improving the photosynthetic efficiency of wheat functional leaves is crucial for grain weight accumulation [45]. Leaf photosynthesis is mainly characterized by stomatal conductance, transpiration rate, net photosynthetic rate and intercellular CO_2_ concentration [46]. Previous studies have demonstrated that combined incorporation of green manure and wheat straw significantly increases soil nitrogen content, mitigates nitrogen loss, and improves the uptake of chemical nitrogen fertilizer by winter wheat, compared with their separate application [47]. Therefore, under reduced nitrogen application, the combined returning of green manure and wheat straw exerts a greater promoting effect on the photosynthetic characteristics of spring wheat than the sole returning of either material, which is consistent with the findings of the present study (Figure 2). Previous studies have demonstrated that green manure incorporation increases leaf SPAD values in crops [4]. Li et al. [48] reported that straw incorporation also increased SPAD values in wheat leaves. The present study demonstrated that all organic material returning treatments significantly increased leaf SPAD values, with combined returning exhibiting the most pronounced effect. This may be attributed to the fact that residue incorporation increases soil available nitrogen, which promotes root growth, improves plant nitrogen transformation efficiency, and regulates chlorophyll synthesis and metabolism, ultimately elevating leaf SPAD values. Random forest analysis revealed that intercellular CO_2_ concentration, *korA* and soil organic carbon were the key factors driving yield (Figure 6). This indicates that Ci regulates the rate of photosynthetic carbon fixation and acts as a critical determinant of crop yield.

### 4.2. Effects of Organic Material Returning on Soil Organic Carbon

Soil organic carbon is a core indicator for evaluating soil fertility and agricultural productivity, and plays a vital role in regulating soil quality and ecological function [49]. Strategies for enhancing soil organic carbon primarily focus on increasing carbon inputs, reducing carbon losses, or integrating both approaches [50]. The present results show that organic material returning effectively increases soil organic carbon content and stocks (Figure 3a,b), which is in agreement with the findings of Zhang et al. [51]. Organic residues serve as effective organic substrates that supply substantial carbon and nutrients to soil after incorporation. This process alters the living environment of soil microorganisms, and increases the relative abundance of microbial communities and functional genes, and thereby stimulates bacterial proliferation and modulates bacterial community structure. The optimized microbial community further accelerates residue decomposition and nutrient release, ultimately facilitating soil carbon accumulation.

### 4.3. Effects of Organic Material Returning on Soil Microorganisms

Soil ecological functions are largely mediated by microbial communities, with bacteria and fungi as the core components. Together with other microbes, they drive energy flow and nutrient cycling within soil ecosystems and sustain ecosystem stability. Long-term field experiments conducted in the North China Plain revealed that microbial communities responded to residue incorporation mainly via shifts in community structure, rather than pronounced changes in diversity [52]. This finding is consistent with our results (Figure S2). Residue incorporation can enhance the diversity of soil bacterial communities to a certain degree [53], and this study also shows that returning increased bacterial community diversity (Figure S2). Plant residues incorporated via returning practices exhibit high chemical heterogeneity. On the one hand, they provide diverse carbon sources for soil bacteria with distinct metabolic functions. On the other hand, residue input modulates soil aggregate structure, porosity and local oxygen concentration, forming diverse soil microhabitats. These changes alleviate competitive exclusion among dominant bacterial taxa and ultimately improve soil bacterial community diversity. Straw incorporation alters soil habitats through exogenous substrate input, thereby driving the overall succession of bacterial communities from an oligotrophic to a copiotrophic trophic strategy [54,55]. In this study, the combined green manure and wheat straw treatment (GW) markedly enriched the phylum Chloroflexi. Green manure alone (G) and wheat straw alone (W) selectively enriched nitrogen-adapted microbiota and recalcitrant carbon-degrading bacteria, respectively. By contrast, the nitrogen-only treatment (N) was predominated by oligotrophic taxa (Figure 4). Green manure supplies readily decomposable organic matter with high nitrogen content, which selectively favors nitrogen-loving r-strategists. Wheat straw is abundant in cellulose and lignin, thereby enriching microbes capable of degrading recalcitrant carbon compounds. The GW treatment creates a carbon–nitrogen coupled microenvironment with balanced energy input; it supplies diverse carbon sources and relieves nitrogen limitation, and consequently enriches microbial groups that decompose organic matter and adapt to moderate carbon and nitrogen conditions. Long-term absence of fresh organic matter input under the N treatment sustains a community dominated by oligotrophic bacteria.

Studies have demonstrated that carbon-sequestering microorganisms are ubiquitous across diverse ecological environments. At present, molecular biological techniques are primarily applied to investigate their abundance, community composition and diversity. Generally, higher abundance of functional genes indicates stronger microbial carbon sequestration potential [56]. Soil bacterial alpha diversity indices serve as key indicators for evaluating bacterial community diversity and ecosystem stability. Previous studies have demonstrated that green manure and combined residue returning strongly stimulate chemolithoautotrophic processes (represented by nitrification) via the input of labile carbon sources. These processes provide sufficient energy for functional microorganisms, promote the proliferation and metabolic activity of key enzymes encoded by carbon-sequestration-related genes, and ultimately significantly increase the relative abundance of these functional genes [57]. As green manure incorporation amount increases (Figure S13), the alpha diversity of the *cbbS* gene also increases (Figure S7). This improvement can be attributed to the labile and readily degradable organic carbon inputs derived from green manure, which also improve soil aeration and establish heterogeneous microhabitats for carbon-sequestering microorganisms harboring distinct *cbbS* genotypes. Meanwhile, organic acids, flavonoids, and nitrogen nutrients released during green manure decomposition effectively regulate rhizosphere soil redox conditions and nutrient substrate competition. These effects alleviate the growth limitation of microbial communities imposed by single carbon sources, facilitate the coexistence of autotrophic and heterotrophic microbial taxa, and ultimately enhance the community richness of soil *cbbS* functional genes. Straw incorporation significantly decreased the Simpson index of the *ppdK* gene (Figure S7). The organic carbon substrates introduced by straw returning confer a strong competitive advantage on *ppdK*-harboring microbial taxa, which reduces the evenness of the functional community distribution and ultimately decreases the Simpson diversity index. Crop residue incorporation substantially alters soil bacterial community structure and elevates the relative abundances of Bacteroidetes, Proteobacteria and Gemmatimonadetes [58]. The research of Li et al. [55] shows that straw returning reduced the relative abundance of Actinobacteria. In this study, the dominant genera varied across different carbon-fixing functional gene communities among treatments. Specifically, the *rbcL* community was predominated by Brady rhizobium; the *korA* and *ppdK*/*sdhA* communities were dominated by *Nocardioides*; the *por* community was dominated by unclassified_c_Candidate_Binatia; the *cbbS* community was predominated by Amari coccus; and the *accA* and *gapA* communities were primarily dominated by *Flavobacterium* (Figure S10). These findings indicate that in the heterogeneous microenvironments constructed by straw and green manure returning, the dominant host genera of different carbon metabolism genes are determined by the precise matching of microbial metabolic pathways and ecological niches. Specifically, Brady rhizobium primarily contributes to the Calvin cycle. *Nocardioides* possesses both microaerobic and aerobic metabolic capacities, regulating the *korA*-mediated reductive TCA cycle under fluctuating redox conditions, the *ppdK*-dependent carbon cataplerosis process, and the *sdhA*-linked connection between the TCA cycle and electron transport chain. unclassified_c_Candidate_Binatia adapts to anaerobic microhabitats and drives TCA-pathway-based carbon fixation via *por*. Amari coccus utilizes facultative photoheterotrophic characteristics to maintain high *cbbS* expression in topsoil. As typical aerobic heterotrophic bacteria, *Flavobacterium* promotes *accA*-regulated fatty acid synthesis for carbon storage during the vigorous decomposition stage of organic residues and enhances *gapA*-mediated glycolysis to sustain energy supply, thereby dominating the hosts of *accA* and *gapA*. The specific correspondence between functional genes and microbial genera reveals the microscopic mechanism by which organic returning diversifies soil carbon cycling processes. Straw and combined straw–green manure significantly increase the abundance of carbon fixation and carbon metabolism functional genes. Treatments W and G exhibited higher average clustering coefficients and shorter average path lengths in microbial co-occurrence networks, with uncultured taxa belonging to Rokubacteriota and Dormibacterota identified as the dominant bacterial groups (Figure S11). This suggests that straw and green manure returning yield compact microbial co-occurrence networks characterized by intensive local connections and rapid systemic responses. The microbial communities under these treatments exhibit robust ecological collaboration, along with efficient material and information exchange. The dominant uncultured taxa affiliated with Rokubacteriota and Dormibacterota possess strong capacities for extracellular polysaccharide synthesis and quorum-sensing regulation, enabling the formation of tightly connected functional modules via intensive physical contact and metabolic. Furthermore, their oligotrophy-adapted transmembrane transport systems facilitate efficient resource transfer across distinct network modules, shortening the average path length while maintaining high local clustering. Such topological properties ultimately promote synergistic improvements in soil carbon sequestration function. Compared with the N treatment, the GW treatment exhibited a smaller network diameter, shorter average path length, and lower average degree, with Saccharimonadales identified as the dominant microbial order (Figure S11). These results indicate that combined straw and green manure returning simplifies microbial co-occurrence connections while establishing more direct information transfer pathways among network nodes, forming an overall compact topological structure. As a typical syntrophic symbiotic taxon, Saccharimonadales adapts to the carbon- and nitrogen-rich microenvironments created by combined residue returning. It forms highly specific short-distance metabolic chains by relying on metabolic overflow products from other microbial groups. This niche specialization effectively shortens the functional distance between microbial taxa and reduces network connection density and average degree. The GW treatment exhibited a higher aggregation degree (Figure S11), indicating a more homogeneous intra-group community structure. By integrating both labile and recalcitrant carbon sources, GW sustains a broader spectrum of microbial functional groups. Intensive microbial interactions further construct a functionally redundant network with compact internal connections and enhanced resistance to external environmental disturbances. Such structural characteristics enable the microbial community to maintain high structural homogeneity and stability, even under spatially heterogeneous soil conditions [59]. The abundance of the *korA* gene harbored by Chloroflexota, Candidatus_Deferrimicrobiota, Candidatus_Margulisbacteriota, and Candidatus_ Lindowiibacteriota differed significantly across treatments (Figure 5). Separate and combined applications of straw and green manure differentially regulate soil C/N ratio, oxygen availability, and organic matter decomposition rates. These alterations reshape substrate supply and metabolic microenvironments for anaerobic taxa such as Chloroflexota, ultimately driving significant treatment-specific differences in *accA* gene abundance. The *ppdK* gene abundance harbored by Actinomycetota, Pseudomonadota, Candidatus_Binatota, Rhodothermota, candidate_division_WOR-3, Candidatus_Tectimicrobiota, and Bdellovibrionota differed significantly among all treatments (Figure 5). These results demonstrated that organic returning does not uniformly activate carbon cataplerotic metabolism across microbial taxa but drives the differential enrichment of the *ppdK* gene within distinct microbial groups, predominantly Actinomycetota and Pseudomonadota. The *gapA* gene abundance of Actinomycetota, Candidatus_Binatota, Candidatus_Methylomirabilota, and Candidatus_Deferrimicrobiota differed significantly across treatments (Figure 5). The distinct molecular structures of returned organic materials impose selective regulation on the glycolytic substrate metabolism and energy capture efficiency of different microbial phyla. Actinomycetota predominantly depend on *gapA*-mediated glycolytic pathways in cellulose- and hemicellulose-enriched microhabitats. In contrast, candidate taxa such as Candidatus_Binatota adapt to localized hypoxic conditions and only partially activate glycolytic metabolism. These divergent responses ultimately result in significant treatment-specific differences in phylum-level *gapA* gene abundance. The abundance of the *rbcL* gene in Actinomycetota differed significantly among treatments (Figure 5). This finding reflects the intraspecific divergence of energy metabolic adaptation strategies within Actinomycetota under organic residue incorporation. The *rbcL* gene abundance of Picozoa differed significantly across treatments (Figure 5). Organic residue incorporation alters the composition of soil dissolved organic carbon and reshapes microbial community structure. These variations further modulate the abundance of bacteria and protist-harbored *rbcL* genes via predation and endosymbiosis, ultimately driving significant treatment-specific differences in *rbcL* functional gene profiles. The *accA* gene abundance of Verrucomicrobiota differed significantly across treatments (Figure 5), indicating the divergent adaptive patterns of this phylum in carbon storage and membrane synthesis metabolism under organic residue incorporation. The *gapA* gene abundance exhibited extremely significant positive correlations with soil organic carbon, net photosynthetic rate, intercellular CO_2_ concentration, stomatal conductance, transpiration rate, SPAD value, and dry matter mass (Figure 6a). This demonstrates that the *gapA* gene facilitates crop dry matter accumulation by synergistically promoting chlorophyll synthesis, improving photosynthetic capacity, and enhancing soil carbon sequestration. The *ppdK* gene abundance showed significant positive correlations with Ci, Tr and SPAD (Figure 6a). This suggests that the *ppdK* gene elevates chlorophyll content, thereby increasing transpiration rate and intercellular CO_2_. Total bacterial abundance was positively correlated with transpiration rate (Tr) (Figure 6a), suggesting that higher bacterial abundance corresponds to greater plant transpiration. Bacteria can modulate stomatal behavior to enhance plant transpiration; in turn, increased transpiration stimulates root exudation, which further facilitates bacterial proliferation. The study region features long winters with low temperatures, and soil microbial activity remains at a minimum for a substantial fraction of the year. Winter snow cover modulates soil temperature and moisture regimes, and influences freeze–thaw cycles as well as microbial community structure. Straw return may further regulate microbial activity and carbon and nitrogen transformation processes by modifying surface cover and soil hydrothermal conditions. Therefore, when interpreting the effects of straw return treatments on microbial communities and carbon sequestration, the seasonal constraints imposed by the alpine environment and the potential role of snow cover should be fully considered. This study did not directly monitor the dynamics of winter microbial activity; future investigations should integrate winter soil hydrothermal and microbial observations for further validation. In the no-return control, the observed SOC increase may primarily stem from crop-root-derived carbon inputs. Despite the absence of exogenous organic material return in this treatment, crops continued to grow, with roots, root exudates, stubble, and fine root turnover continuously supplying organic carbon to the soil. Accordingly, no residue return does not equate to zero carbon input. SOC can remain stable or even rise when root carbon inputs approach or exceed SOC mineralization losses.

## 5. Conclusions

Straw return improved bacterial evenness. Combined return increased β-diversity, enriched the bacterial phylum Chloroflexi and fungal genus *Fusarium*, and formed a functionally complementary microbial community. Under green manure return, the Chao1 index of the *cbbS* gene was significantly higher than that under the no-return treatment, accompanied by increased crop yield. Straw return reduced the Simpson index of the *gapA* gene. Combining straw and green manure return integrated the merits of individual straw and green manure amendments, achieving the largest increases in spike number, grains per spike, 1000-grain weight and grain yield. Meanwhile, this practice shortened network diameter and average path length and decreased average degree, shifting the carbon-sequestering microbial network from a loose, expanded architecture to a compact modular structure. All treatments increased net photosynthetic rate, stomatal conductance, transpiration rate, chlorophyll content and soil organic carbon stock, while reducing intercellular CO_2_ concentration.

This study elucidates the mechanisms of carbon sequestration and yield promotion under combined residue return. Nevertheless, several limitations remain unanswered: whether continuous field residue return drives soil organic carbon toward its saturation threshold, and whether yield-promoting effects diminish once carbon saturation is reached. All functional gene analyses in this work relied on relative abundance data without absolute quantification, which prevents us from distinguishing whether treatment-induced differences stem from shifts in microbial community structure or variations in total microbial biomass. Furthermore, data for photosynthetic and soil-carbon-fixation functional genes were limited to a single growing season. Future research should adopt multi-year field experiments to identify the optimal duration and organic input threshold for combined return. Absolute gene quantification should also be performed to disentangle the drivers underlying variations in gene abundance. Future research will combine indicators including NH_4_^+^-N, NO_3_^−^-N, and nitrification- and denitrification-related functional genes or enzyme activities to further elucidate the mechanisms of nitrogen transformation in alpine croplands. The biomass of the preceding crop was not directly measured in this study. The absence of direct measurements of crop biomass, root biomass, and root-derived carbon inputs constrained our ability to fully elucidate the observed SOC dynamics. Future studies should directly quantify these variables to more accurately assess the contribution of root-derived carbon to soil organic carbon.

## Figures and Tables

**Figure 1:**
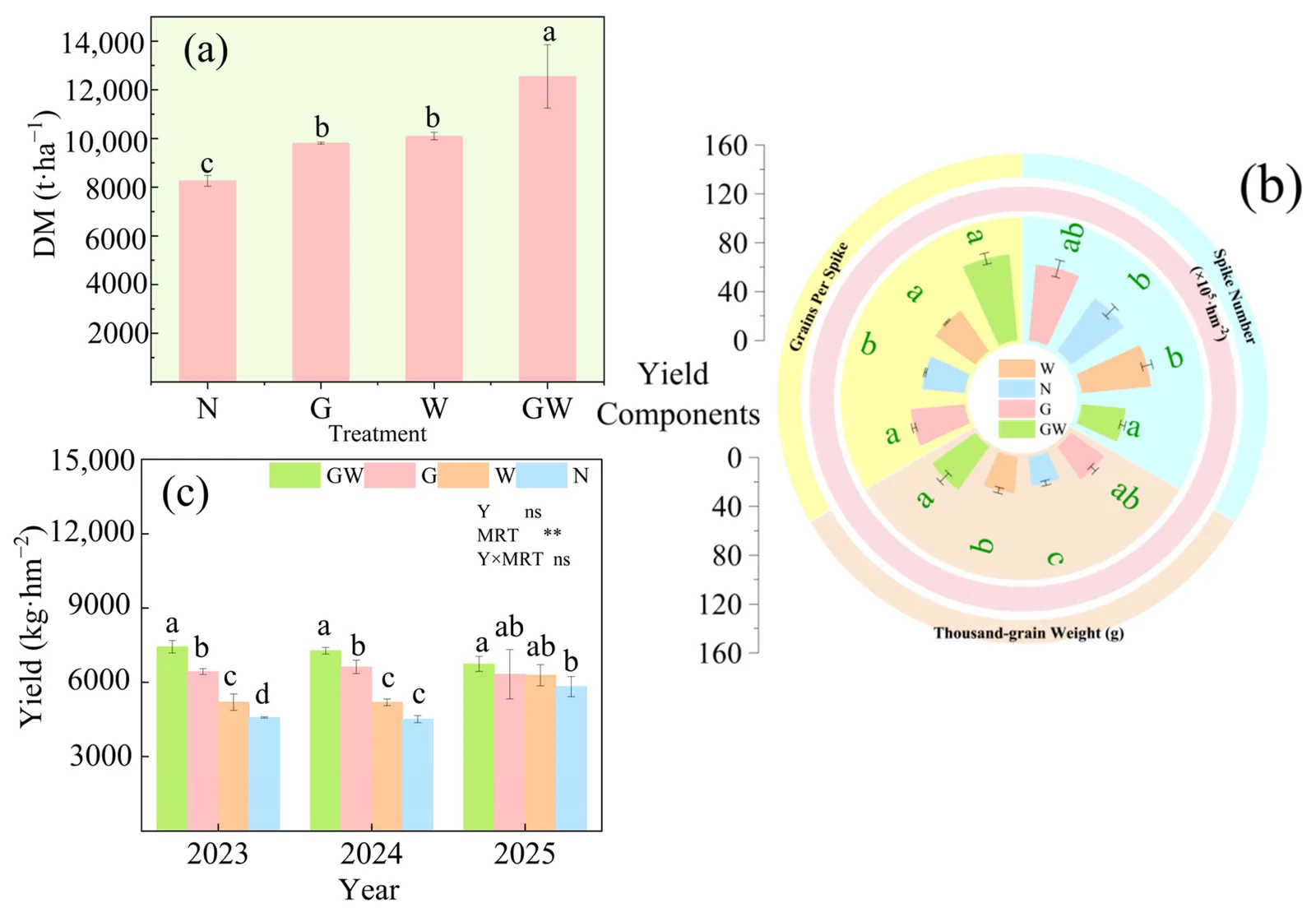
Wheat yield, yield components, and dry matter accumulation under organic residue incorporation. Note: Dry matter and yield components were determined in 2025; grain yield was determined from 2023 to 2025. Each plot served as the experimental unit, with three replicates (*n* = 3). Data are presented as mean ± SD. (**a**) Dry matter accumulation; (**b**) Yield components; (**c**) Grain yield. ** means extremely significant correlation at the 0.01 level, ns means no significant difference. Different lowercase letters indicate significant differences among organic material treatments (*p* < 0.05).

**Figure 2:**
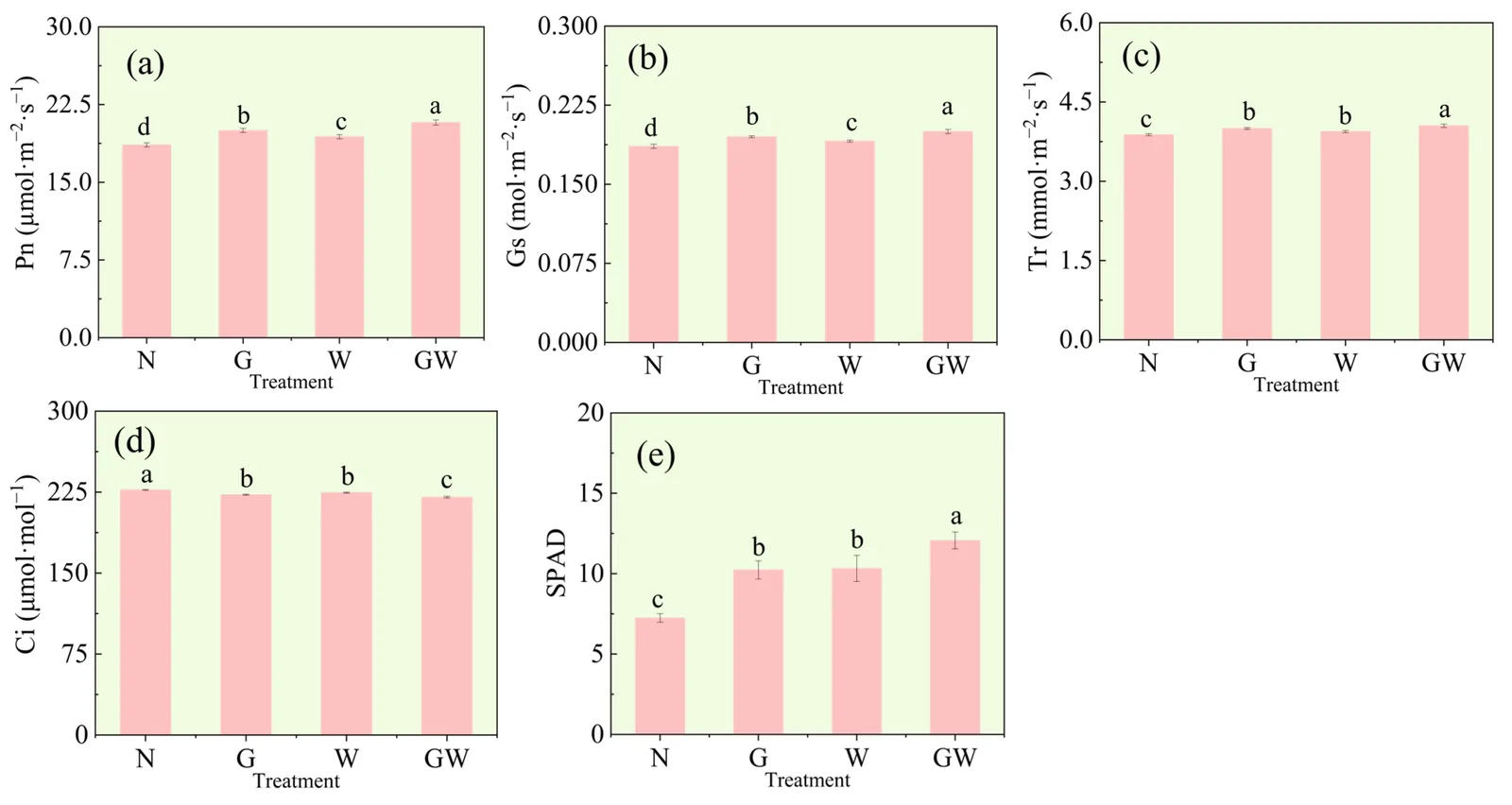
Leaf photosynthetic physiological indicators under material returning. Note: Different lowercase letters indicate significant differences among organic material treatments (*p* < 0.05). SPAD indicates chlorophyll content, Pn indicates net photosynthetic rate, Tr indicates transpiration rate, Gs indicates stomatal conductance, and Ci indicates intercellular CO_2_ concentration. Note: Photosynthetic parameters were measured in 2025. For all indicators, each plot served as the experimental unit with three replicates (*n* = 3). Data are presented as mean ± SD. (**a**) Net photosynthetic rate (Pn) of leaves; (**b**) stomatal conductance (Gs) of leaves; (**c**) transpiration rate (Tr) of leaves; (**d**) intercellular CO_2_ concentration of leaves; (**e**) chlorophyll content.

**Figure 3:**
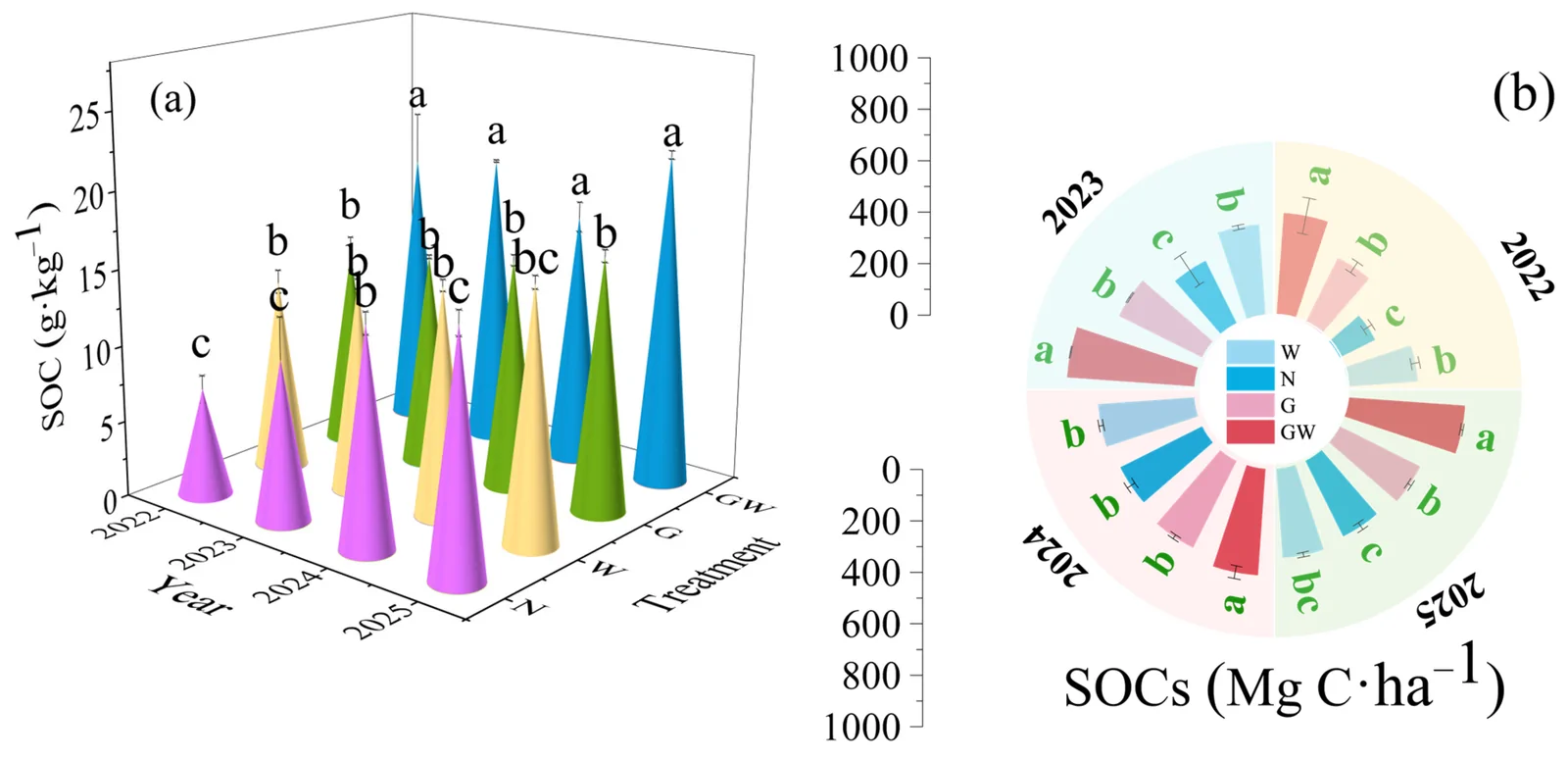
Soil organic carbon content and stock under organic residue incorporation. Note: Different lowercase letters indicate significant differences among organic material treatments (*p* < 0.05). SOC: soil organic carbon; SOCs: soil organic carbon stock. (**a**) Organic carbon content; (**b**) Organic carbon stock.

**Figure 4:**
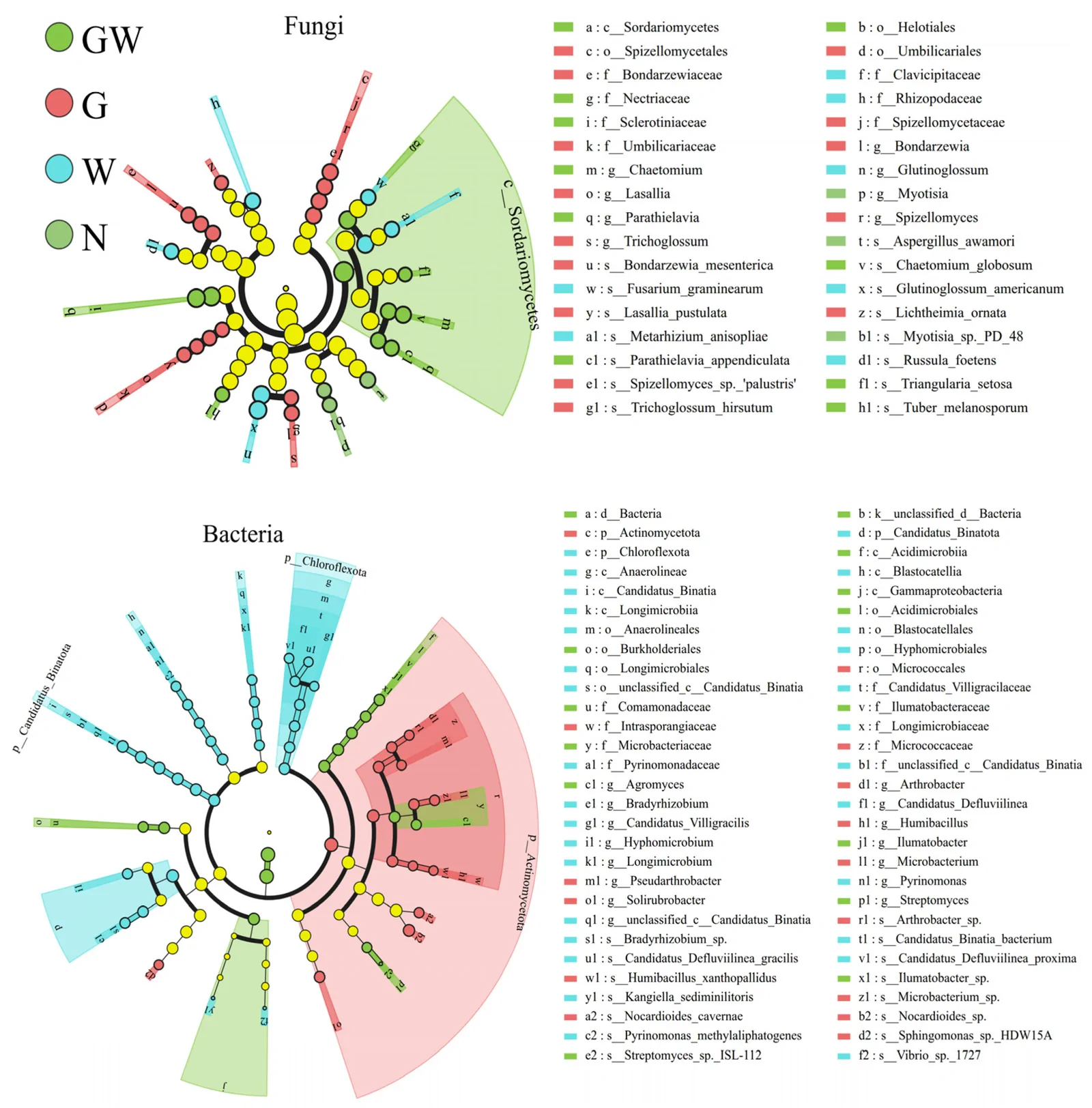
Differential analysis of bacteria and fungi.

**Figure 5:**
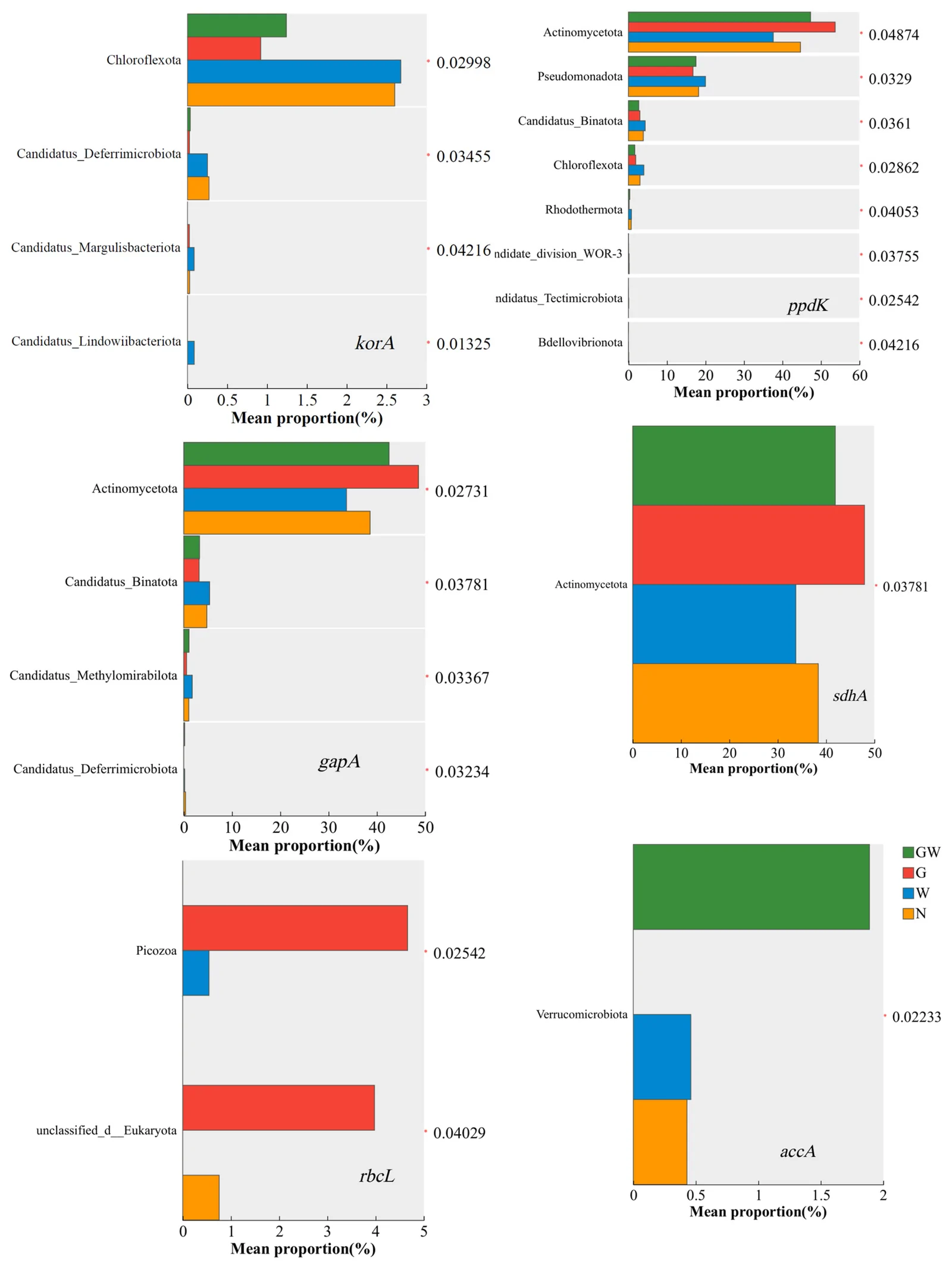
Differential abundance of microbial taxa associated with carbon-metabolism-related functional genes under different organic residue treatments.

**Figure 6:**
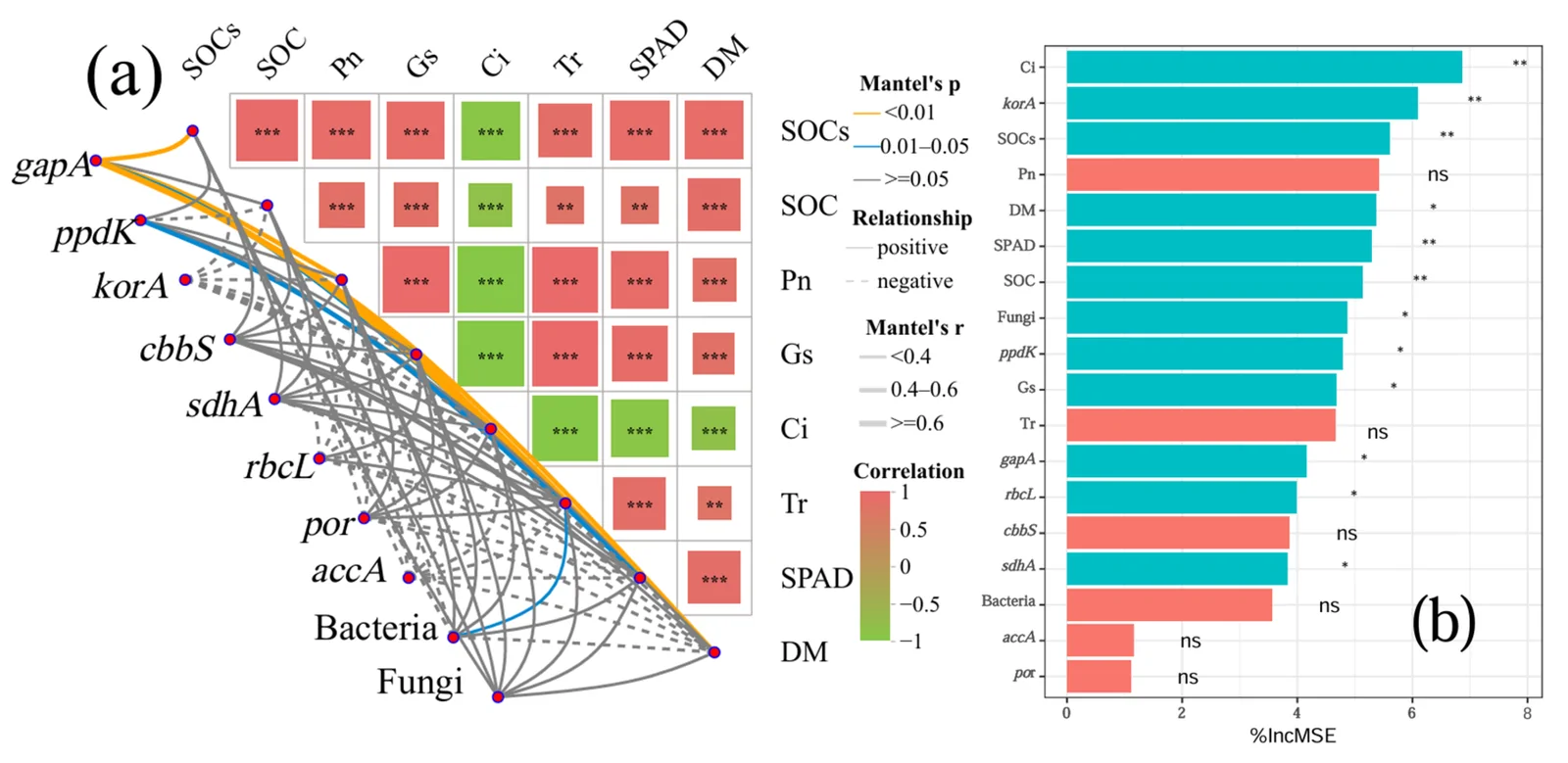
Correlations of bacterial and fungal communities with carbon fixation and carbon metabolism functional genes and yield. Note: DM: dry matter accumulation; SOC: soil organic carbon; SOCs: soil organic carbon stock. SPAD indicates chlorophyll content, Pn indicates net photosynthetic rate, Tr indicates transpiration rate, Gs indicates stomatal conductance, and Ci indicates intercellular CO_2_ concentration. *** indicates an extremely significant positive correlation at the 0.001 level, ** indicates an extremely significant correlation at the 0.01 level, * indicates a significant correlation at the 0.05 level, and ns indicates no correlation. (**a**) presents Mantel tests linking soil physicochemical variables and functional gene abundances. (**b**) identifies the primary driving factors controlling yield.

**Table 1:** Physicochemical properties of tested brown soil.

pH	SOC (g·kg^1^)	TN (g·kg^−1^)	AP (mg·kg^−1^)	AK (mg·kg^−1^)
8.5	19.7	1.2	13.3	115.7

## Data Availability

The original contributions presented in this study are included in the article. Further inquiries can be directed to the corresponding author.

## Supplementary Materials

The following supporting information can be downloaded at: https://www.mdpi.com/article/10.3390/plants15192909/s1, Figure S1 Distribution of Precipitation and Temperature during the Whole Growth Period of Spring Wheat, Figure S2 Soil bulk density under different residue return treatments, Figure S3 Changes in bacterial and fungal diversity, Figure S4 NMDS analysis of bacterial and fungal communities, Figure S5 Changes in the abundance of bacteria and fungi, Figure S6 Changes in community composition of bacteria and fungi, Figure S7 Co-occurrence network analysis of bacterial and fungal communities, Figure S8 Changes in diversity of carbon sequestration genes, Figure S9 NMDS analysis of bacterial and fungal communities, Figure S10 Changes in the abundance of carbon sequestration genes, Figure S11 Changes in community composition of carbon sequestration genes, Figure S12 Co-occurrence network analysis of carbon sequestration gene communities, Figure S13 Green manure biomass yield, Table S1 Network topological characteristics of key functional communities of bacteria and fungi, Table S2 Network topological characteristics of key functional communities of carbon sequestration genes.
