# Supplementary material for: Combined Return Synergizes Aboveground Photosynthesis and Subsurface Soil Organic Carbon Stock to Boost Spring Wheat Yield in the Qinghai–Tibet Plateau
Source: Plants (Basel). 2026 Sep 23;15(19):2909. doi: 10.3390/plants15192909 (PMC13666649; doi:10.3390/plants15192909)
Supplement: Supplementary file 1 [file plants-15-02909-s001.zip › plants-4504175-supplementary.pdf]

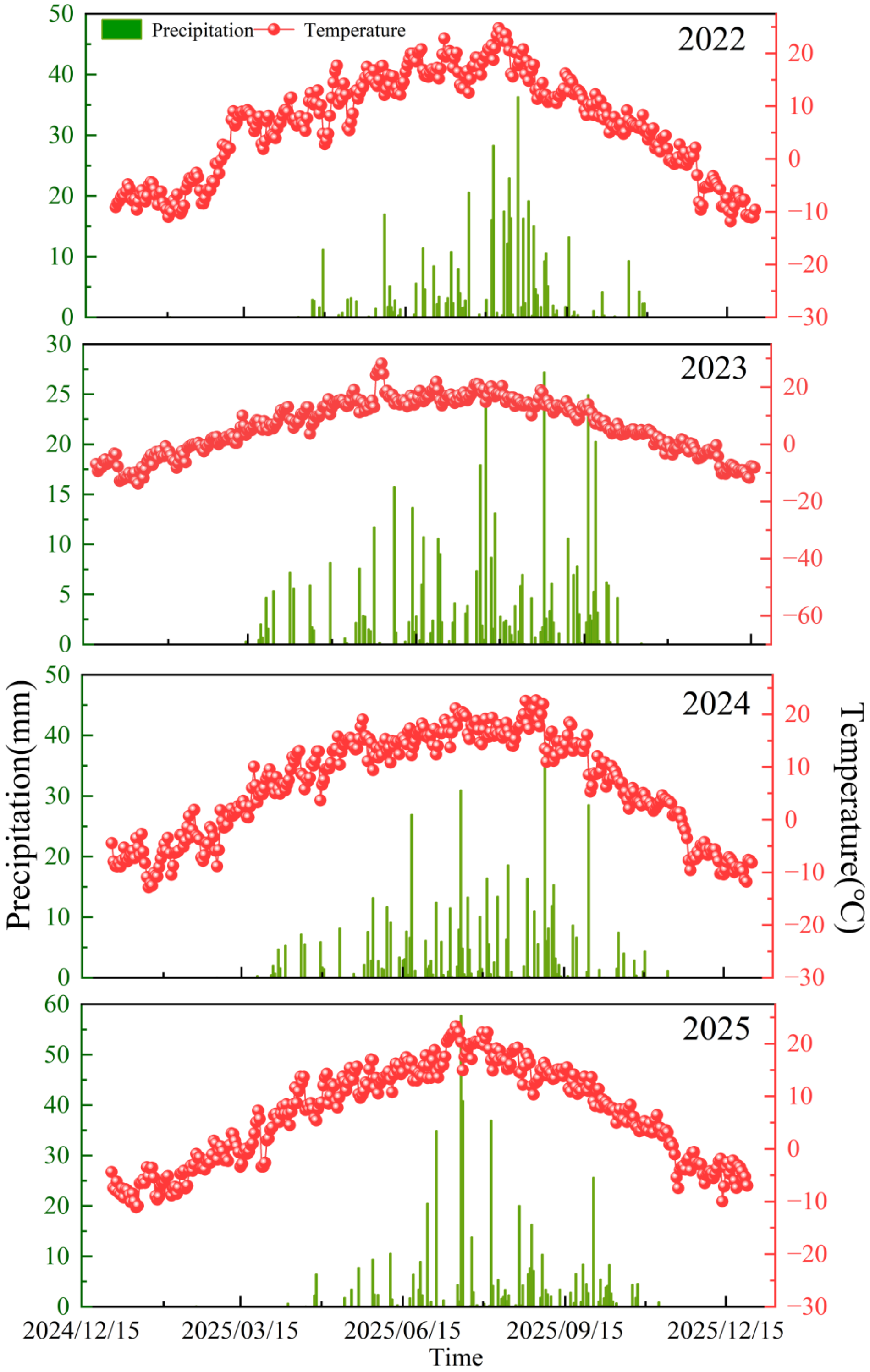

Figure S1 Distribution of Precipitation and Temperature during the Whole Growth Period of Spring Wheat

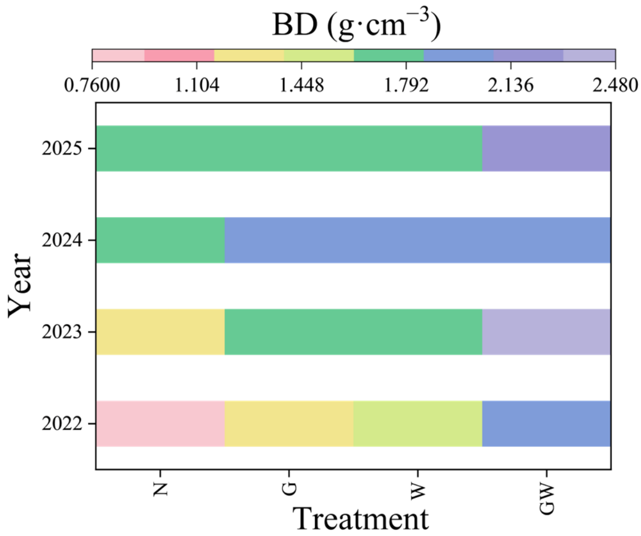

Figure S2 Soil bulk density under different residue return treatments

Note: Soil bulk density was measured from 2023 to 2025. For all indicators, each plot served as the experimental unit with three replicates (n = 3). Data are presented as mean ± SD.

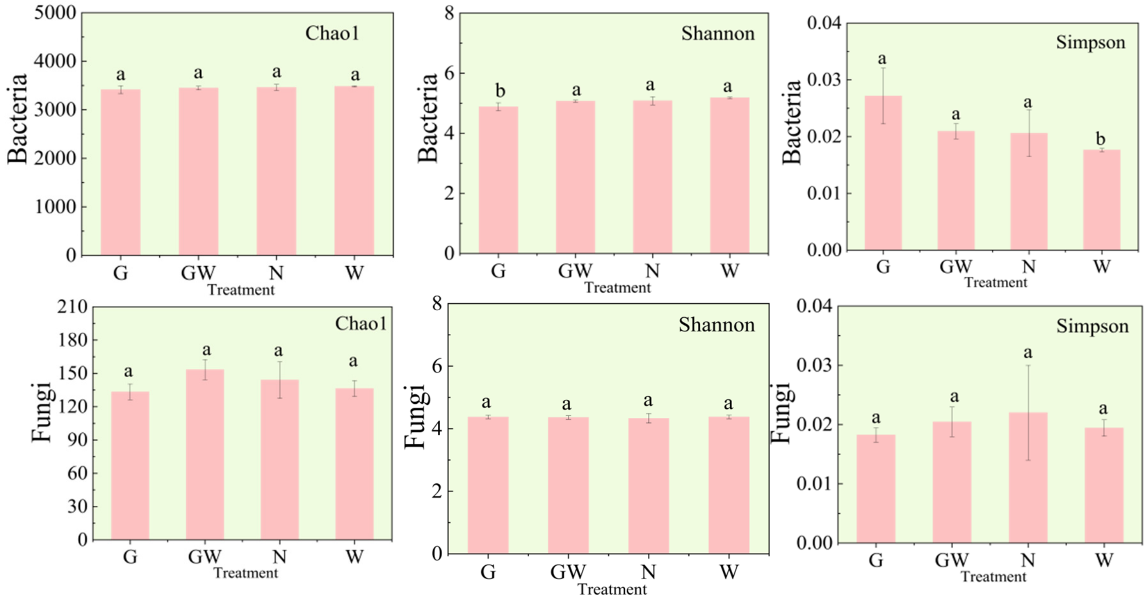

Figure S3 Changes in bacterial and fungal diversity

Note: Gene abundances were measured in 2025. For all indicators, each plot served as the experimental unit with three replicates (n = 3). Data are presented as mean ± SD. Different lowercase letters indicate significant differences among organic material treatments (p< 0.05).

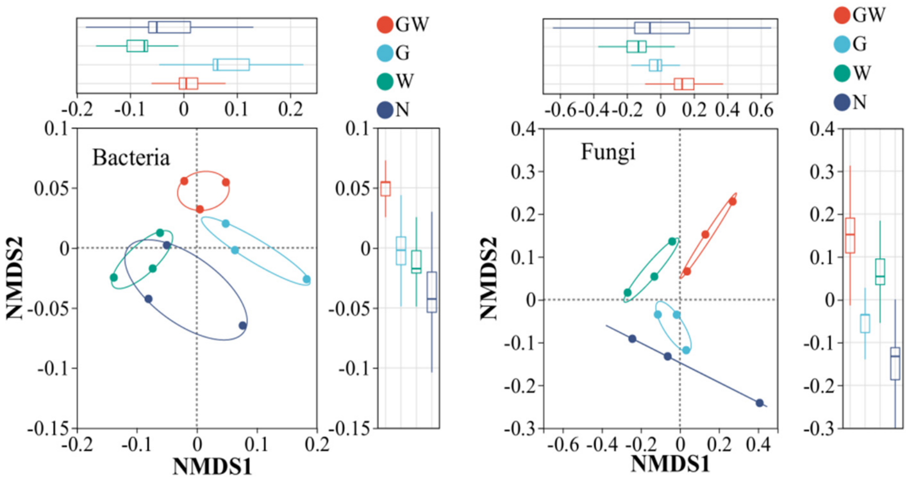

Figure S4 NMDS analysis of bacterial and fungal communities

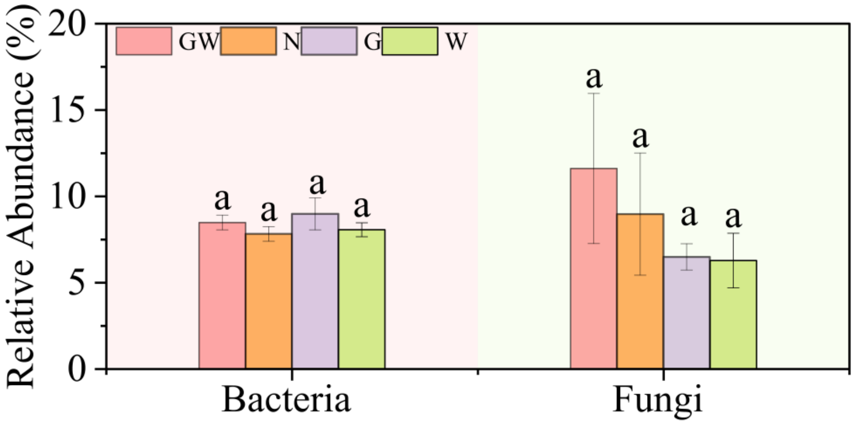

Figure S5 Changes in the abundance of bacteria and fungi

Note: Gene abundances were measured in 2025. For all indicators, each plot served as the experimental unit with three replicates ($n = 3$). Data are presented as mean ± SD. Different lowercase letters indicate significant differences among organic material treatments ($p < 0.05$).

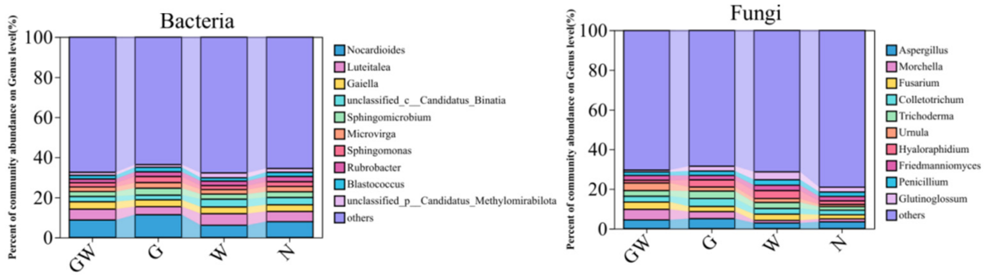

Figure S6 Changes in community composition of bacteria and fungi

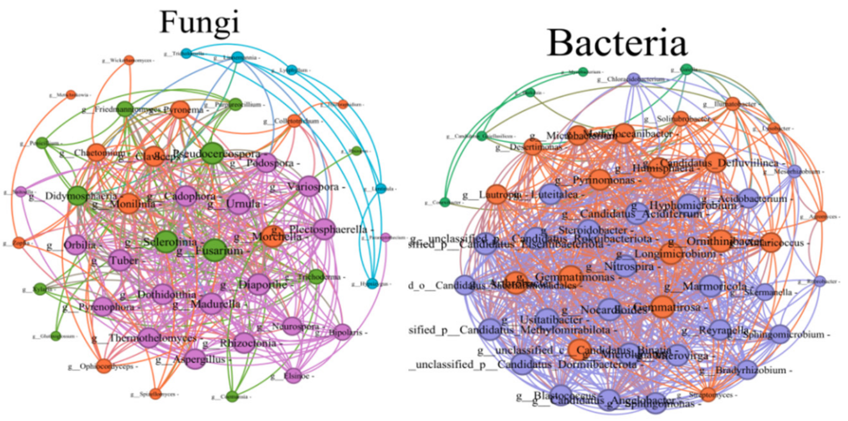

Figure S7 Co-occurrence network analysis of bacterial and fungal communities

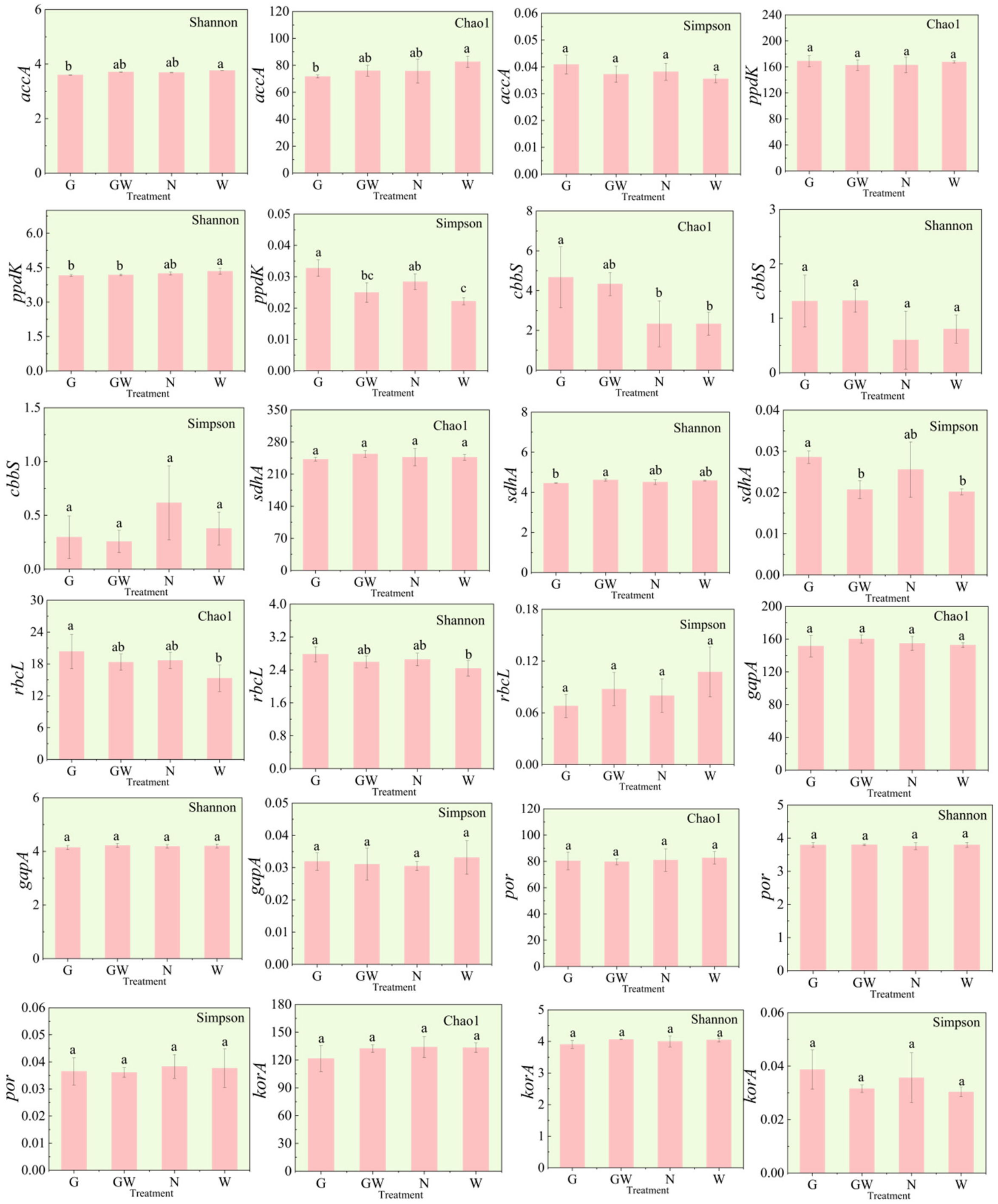

Figure S8 Changes in diversity of carbon sequestration genes

Note: Gene abundances were measured in 2025. For all indicators, each plot served as the experimental unit with three replicates (n = 3). Data are presented as mean ± SD. Different lowercase letters indicate significant differences among organic material treatments (p< 0.05).

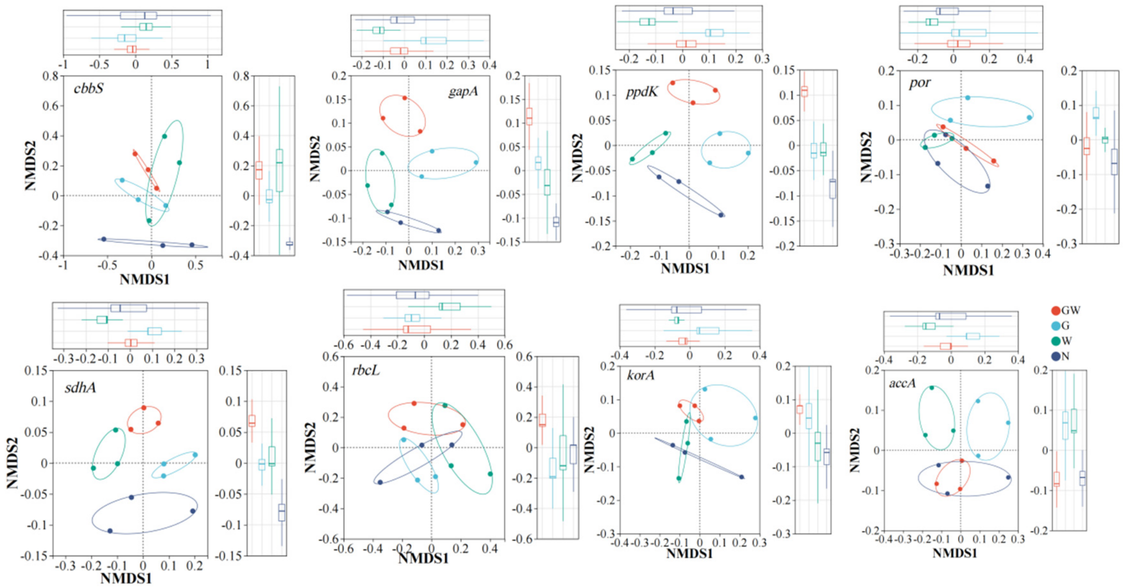

Figure S9 NMDS analysis of bacterial and fungal communities

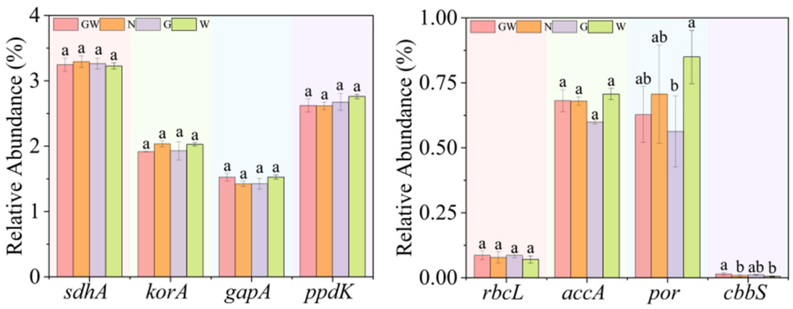

Figure S10 Changes in the abundance of carbon sequestration genes

Note:Different lowercase letters indicate significant differences among organic material treatments (p< 0.05).

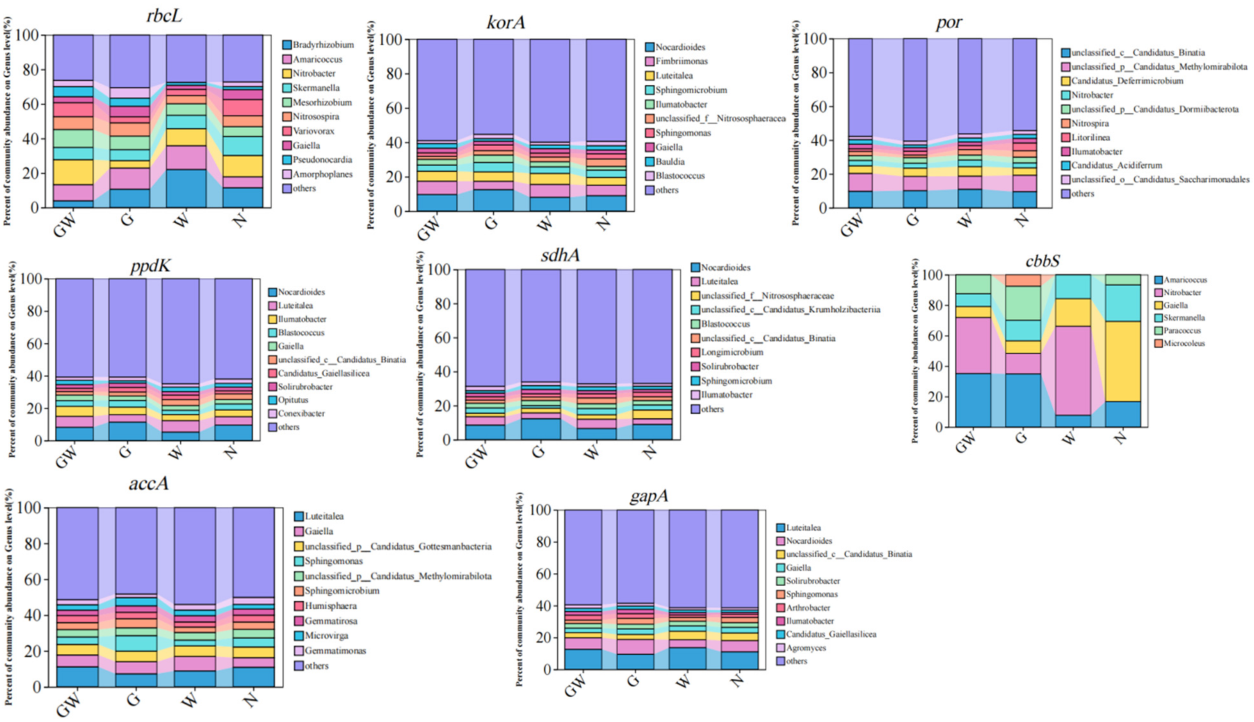

Figure S11 Changes in community composition of carbon sequestration genes

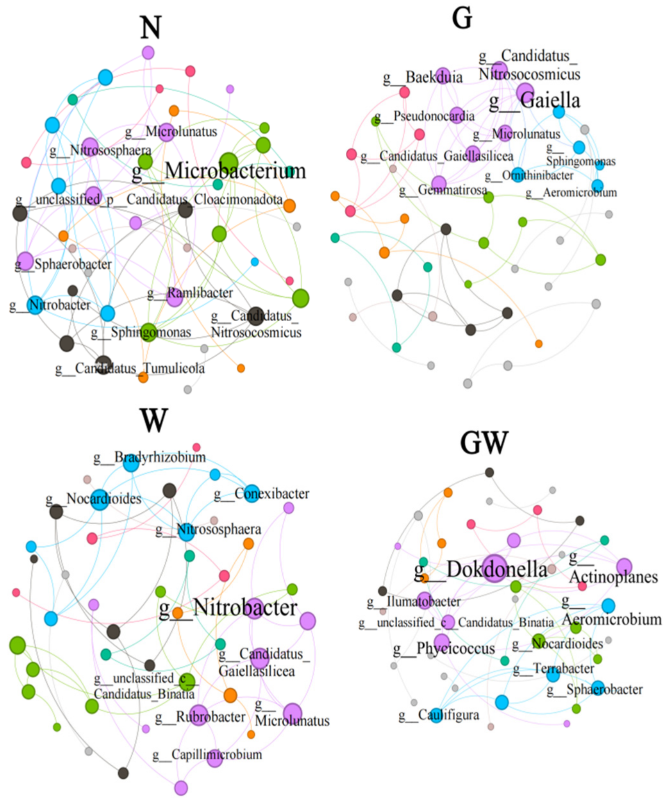

Figure S12 Co-occurrence network analysis of carbon sequestration gene communities

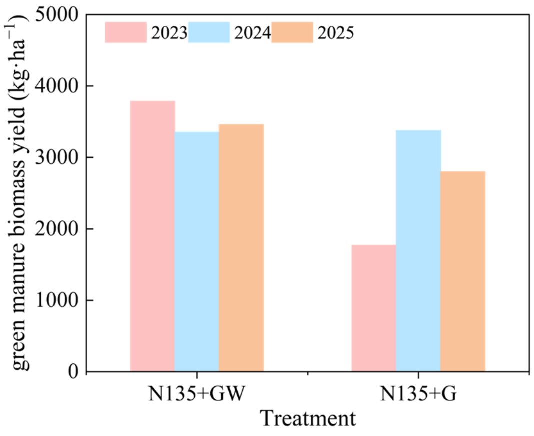

Figure S13 Green manure biomass yield

Note: Green manure yield was measured from 2023 to 2025. For all indicators, each plot served as the experimental unit with three replicates (*n* = 3). Data are presented as mean ± SD.

Table S1 Network topological characteristics of key functional communities of bacteria and fungi

	Average degree	Network diameter	Modularity	Average clustering coefficient	Average path length
Bacteria	26.76	4	0.1	0.777	1.587
Fungi	15.375	6	0.153	0.754	2.168

Table S2 Network topological characteristics of key functional communities of carbon sequestration genes

Treatment	Average degree	Network diameter	Average clustering coefficient	Average path length
G	4.682	7	0.828	2.866
GW	3.565	6	0.762	2.022
W	3.652	4	0.802	1.601
N	4.048	9	0.783	3.273
